# Supplementary material for: Temperature-dependent ligand relocation reveals plasticity of TRPM4 inhibition
Source: bioRxiv. 2026 Jun 9:2026.05.13.724805. Originally published 2026 May 14. Preprint. [Version 2] doi: 10.64898/2026.05.13.724805 (PMC13192758; doi:10.64898/2026.05.13.724805)
Supplement: Supplementary file 1 [file NIHPP2026.05.13.724805v2-supplement-1.pdf]

bioRxiv preprint doi: https://doi.org/10.64898/2026.05.13.724805; this version posted June 9, 2026. The copyright holder for this preprint (which was not certified by peer review) is the author/funder, who has granted bioRxiv a license to display the preprint in perpetuity. It is made available under a CC-BY 4.0 International license.

Supplementary Figures

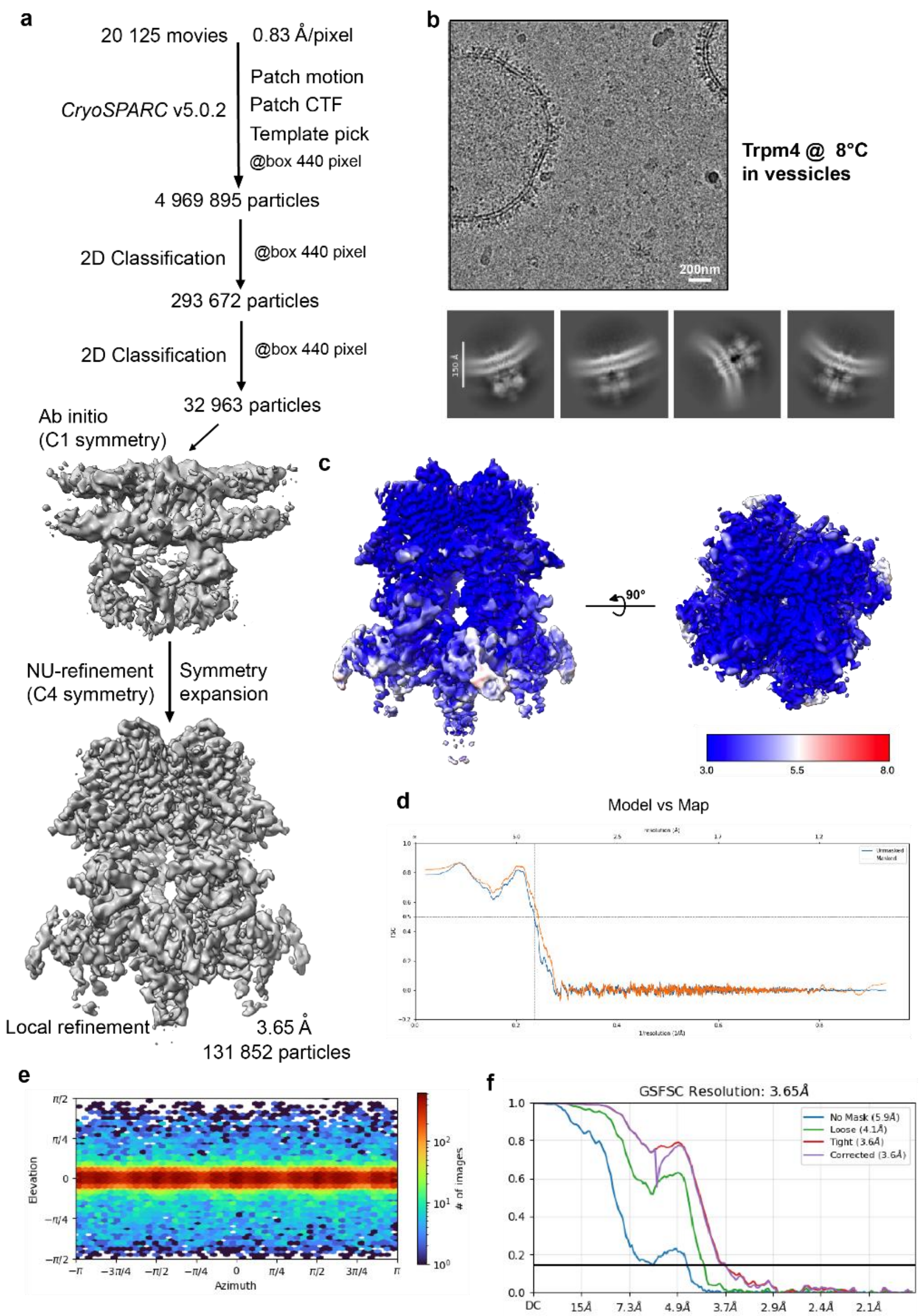

Supplementary Figure 1: **Cryo-EM data processing workflow and map resolution** (a) The image processing workflow of $TRPM4_{vesicle}$. (b) micrograph and 2D classes (c) Local resolution (d) Model vs map FSC curves. (e) particle direction distribution. (f) FSC curves.

bioRxiv preprint doi: https://doi.org/10.64898/2026.05.13.724805; this version posted June 9, 2026. The copyright holder for this preprint (which was not certified by peer review) is the author/funder, who has granted bioRxiv a license to display the preprint in perpetuity. It is made available under a CC-BY 4.0 International license.

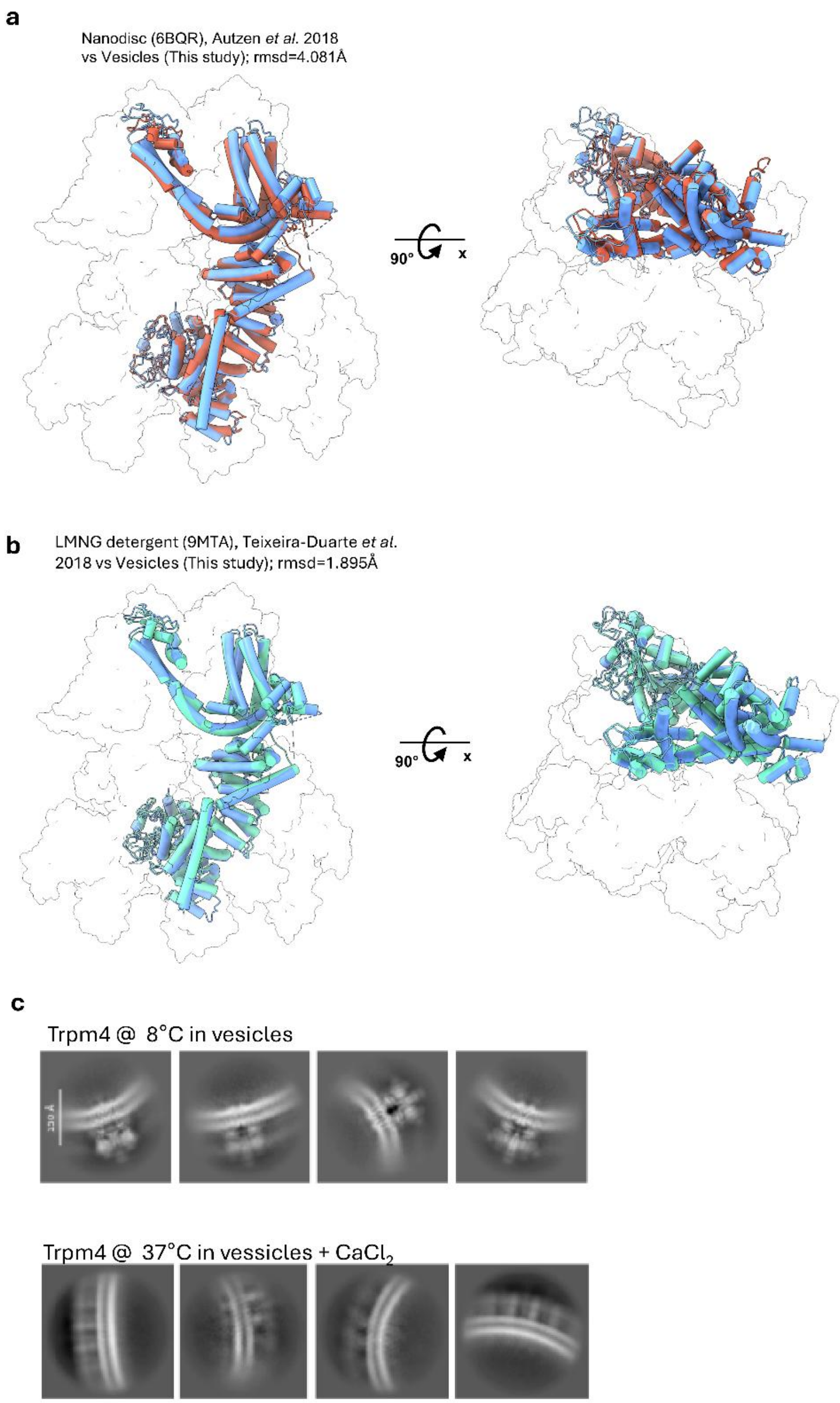

Supplementary Figure 2: **Structural comparison of vesicle-derived TRPM4 with previously reported structures and assessment of vesicle samples prepared at different temperatures. (a)** Superposition of the TRPM4 structure determined in whole-cell vesicles in this study (blue) with the previously reported TRPM4 structure obtained in nanodiscs (PDB

bioRxiv preprint doi: https://doi.org/10.64898/2026.05.13.724805; this version posted June 9, 2026. The copyright holder for this preprint (which was not certified by peer review) is the author/funder, who has granted bioRxiv a license to display the preprint in perpetuity. It is made available under a CC-BY 4.0 International license.

6BQR; salmon) from Autzen *et al* [10]. Side and top views are shown. The two structures superimpose with a root mean square deviation (RMSD) of 4.081 Å. **(b)** Superposition of the TRPM4 structure determined in whole-cell vesicles in this study (blue) with the previously reported TRPM4 structure obtained in LMNG detergent (PDB 9MTA; cyan) from Teixeira-Duarte *et al*. Side and top views are shown [13]. The two structures superimpose with a root mean square deviation (RMSD) of 1.895 Å. **(c)** Representative 2D class averages of TRPM4 particles derived from whole-cell vesicles prepared at different temperatures. Upper panel, vesicles prepared at 8 °C showing well-defined structural features and readily identifiable TRPM4 densities. Lower panel, vesicles prepared at 37 °C in the presence of $CaCl_2$ showing blurred and poorly resolved structural features, indicative of increased particle heterogeneity and preventing high-resolution structure determination.

bioRxiv preprint doi: https://doi.org/10.64898/2026.05.13.724805; this version posted June 9, 2026. The copyright holder for this preprint (which was not certified by peer review) is the author/funder, who has granted bioRxiv a license to display the preprint in perpetuity. It is made available under a CC-BY 4.0 International license.

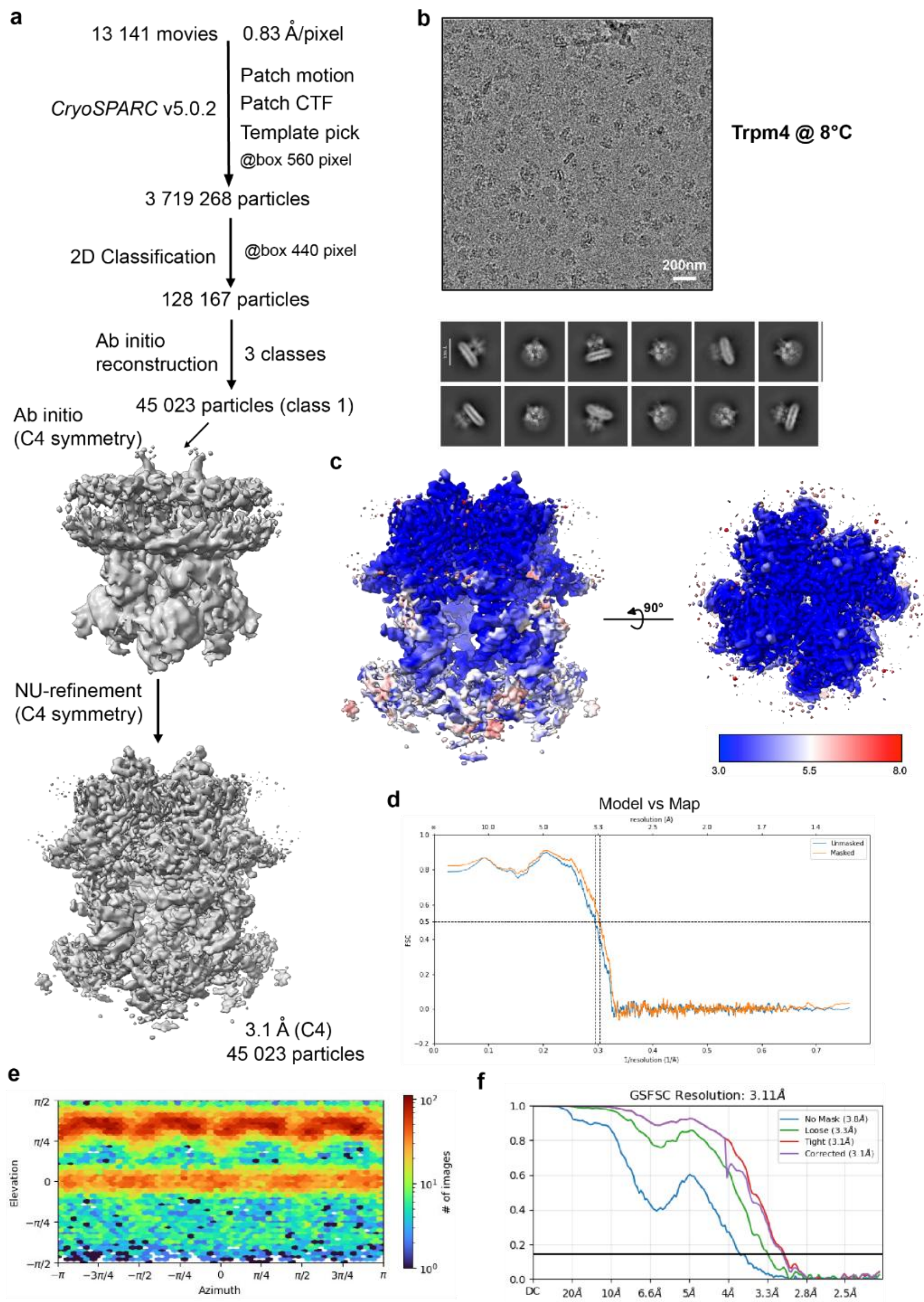

Supplementary Figure 3: **Cryo-EM data processing workflow and map resolution** (a) The image processing workflow of $TRPM4_{apo,\ 8\ °C}$. (b) micrograph and 2D classes (c) Local resolution (d) Model vs map FSC curves. (e) particle direction distribution. (f) FSC curves.

bioRxiv preprint doi: https://doi.org/10.64898/2026.05.13.724805; this version posted June 9, 2026. The copyright holder for this preprint (which was not certified by peer review) is the author/funder, who has granted bioRxiv a license to display the preprint in perpetuity. It is made available under a CC-BY 4.0 International license.

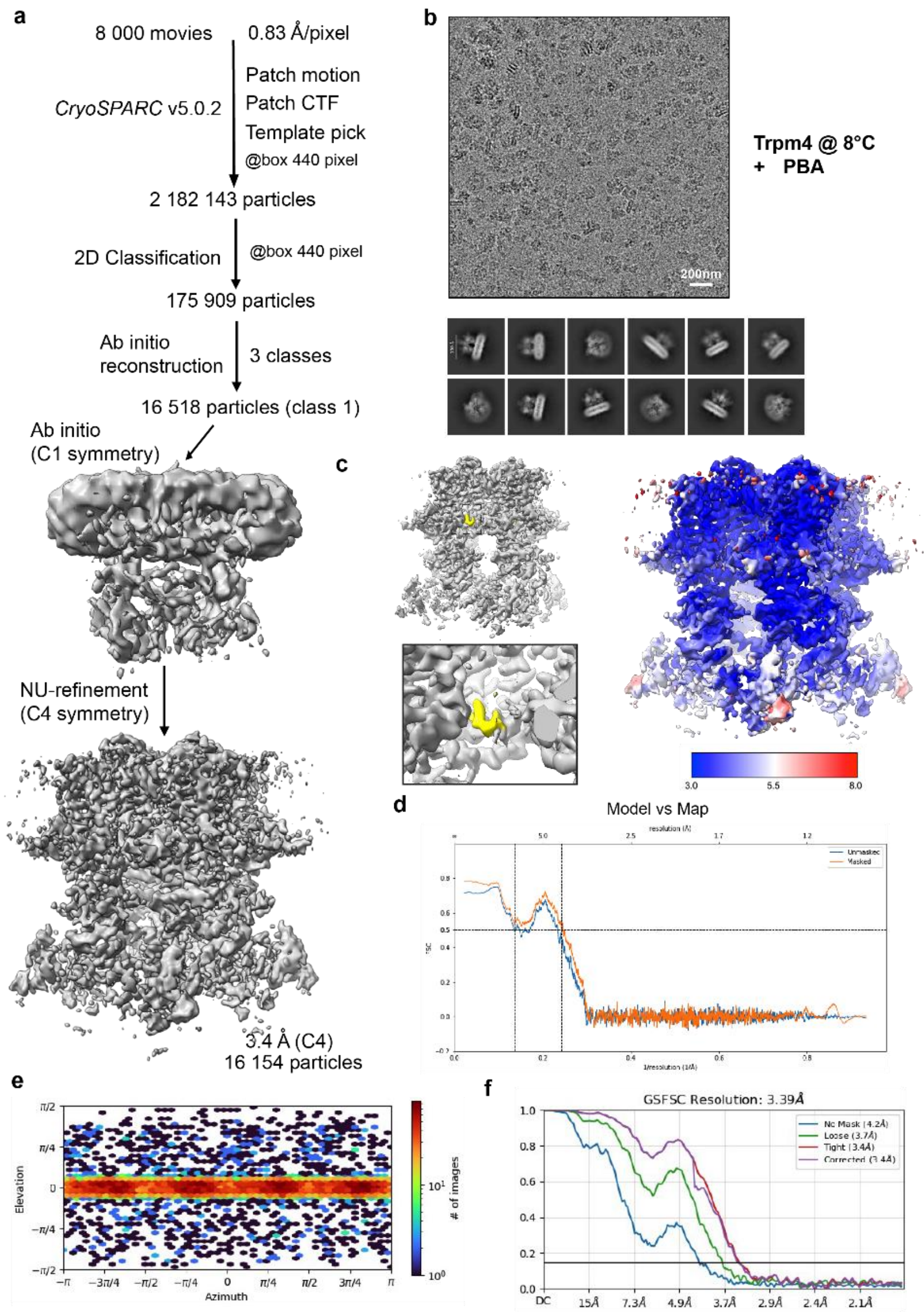

Supplementary Figure 4: **Cryo-EM data processing workflow and map resolution** (a) The image processing workflow of $TRPM4_{PBA,\ 8\ °C}$. (b) micrograph and 2D classes (c) Local resolution and PBA density (yellow) (d) Model vs map FSC curves. (e) particle direction distribution. (f) FSC curves.

bioRxiv preprint doi: https://doi.org/10.64898/2026.05.13.724805; this version posted June 9, 2026. The copyright holder for this preprint (which was not certified by peer review) is the author/funder, who has granted bioRxiv a license to display the preprint in perpetuity. It is made available under a CC-BY 4.0 International license.

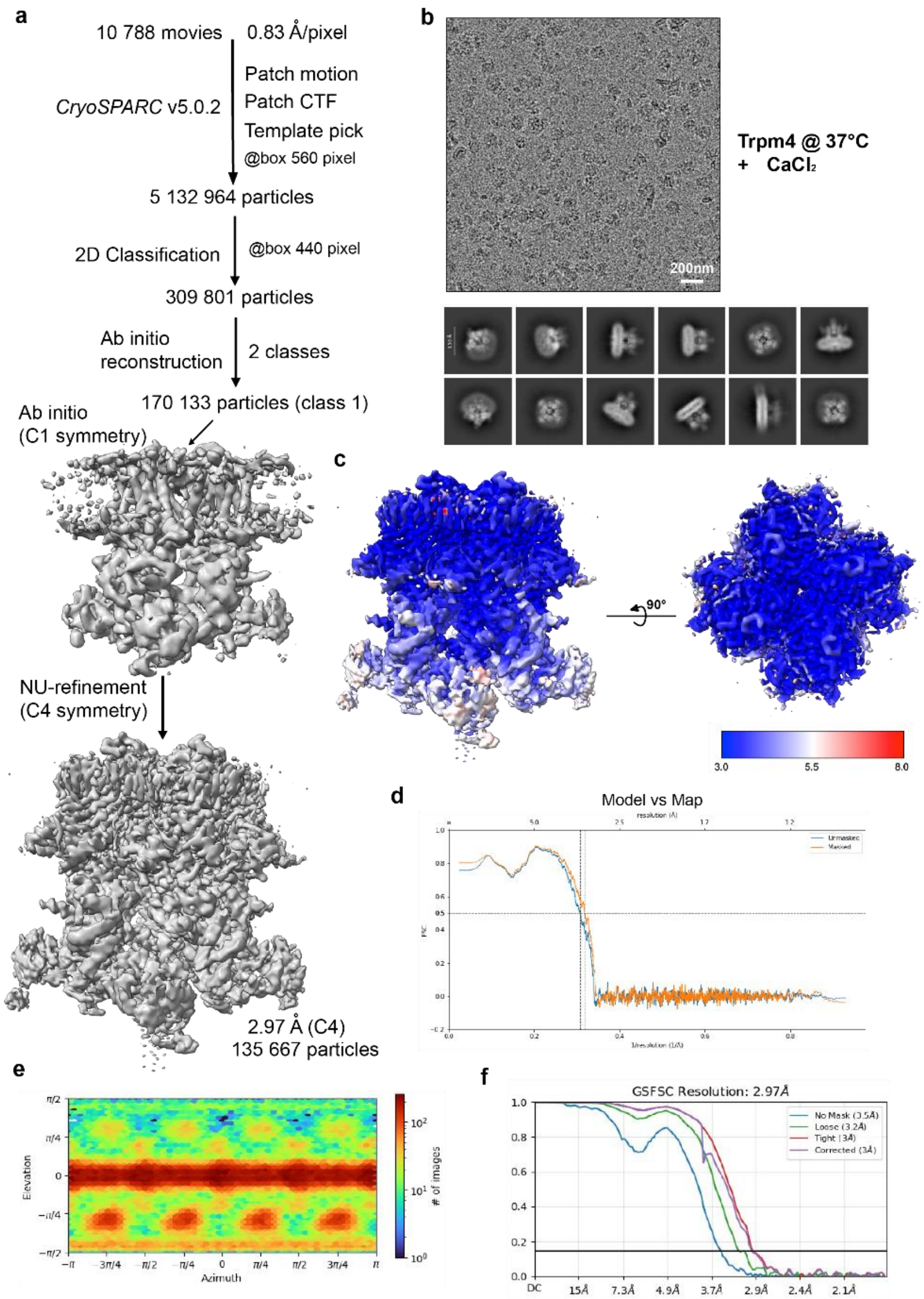

Supplementary Figure 5: **Cryo-EM data processing workflow and map resolution** (a) The image processing workflow of $TRPM4_{apo,\ 37\ °C}$. (b) micrograph and 2D classes (c) Local resolution (d) Model vs map FSC curves. (e) particle direction distribution. (f) FSC curves.

bioRxiv preprint doi: https://doi.org/10.64898/2026.05.13.724805; this version posted June 9, 2026. The copyright holder for this preprint (which was not certified by peer review) is the author/funder, who has granted bioRxiv a license to display the preprint in perpetuity. It is made available under a CC-BY 4.0 International license.

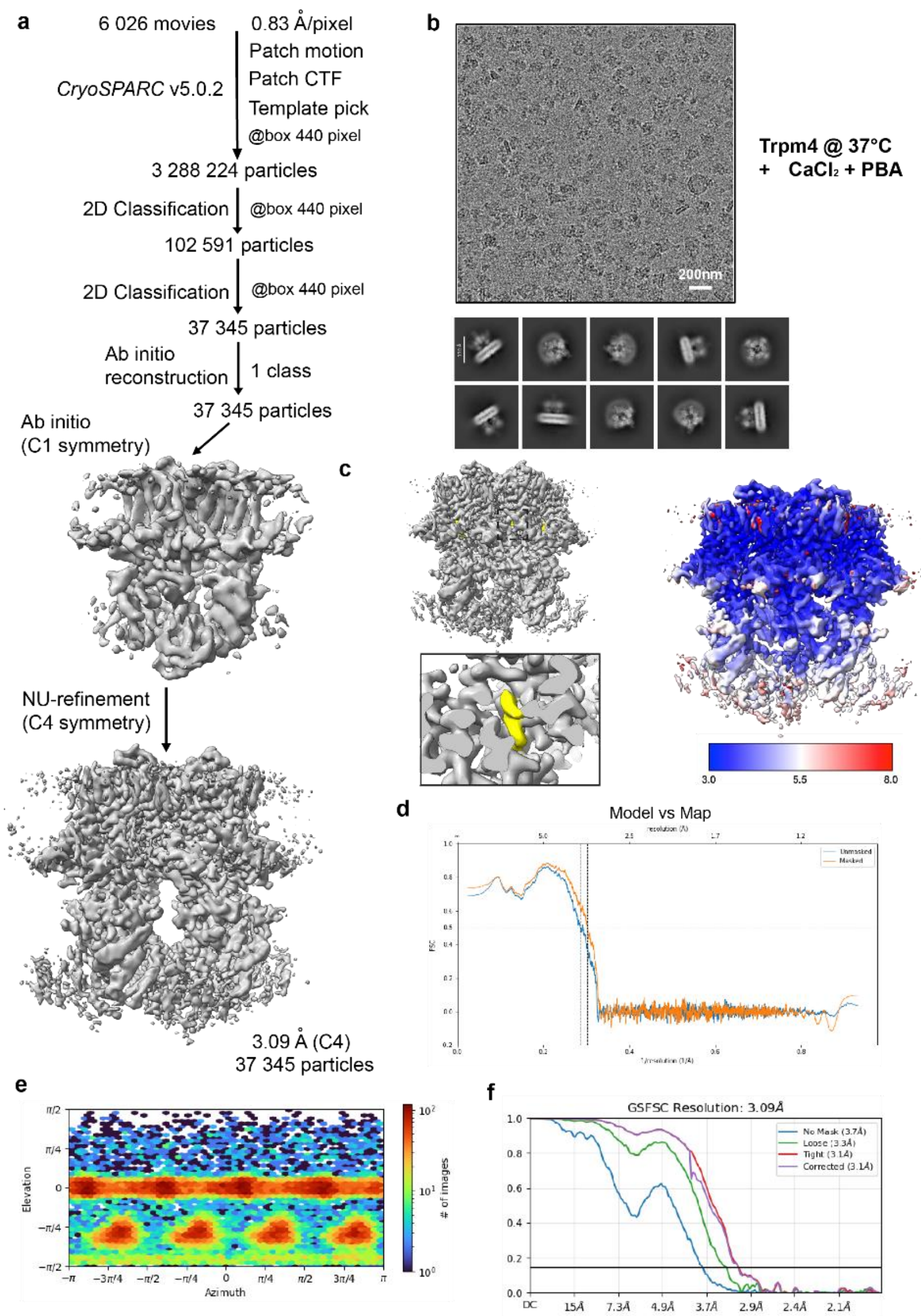

Supplementary Figure 6: **Cryo-EM data processing workflow and map resolution** (a) The image processing workflow of $TRPM4_{PBA,\ 37\ °C}$. (b) micrograph and 2D classes (c) Local resolution and PBA density (yellow) (d) Model vs map FSC curves. (e) particle direction distribution. (f) FSC curves.

bioRxiv preprint doi: https://doi.org/10.64898/2026.05.13.724805; this version posted June 9, 2026. The copyright holder for this preprint (which was not certified by peer review) is the author/funder, who has granted bioRxiv a license to display the preprint in perpetuity. It is made available under a CC-BY 4.0 International license.

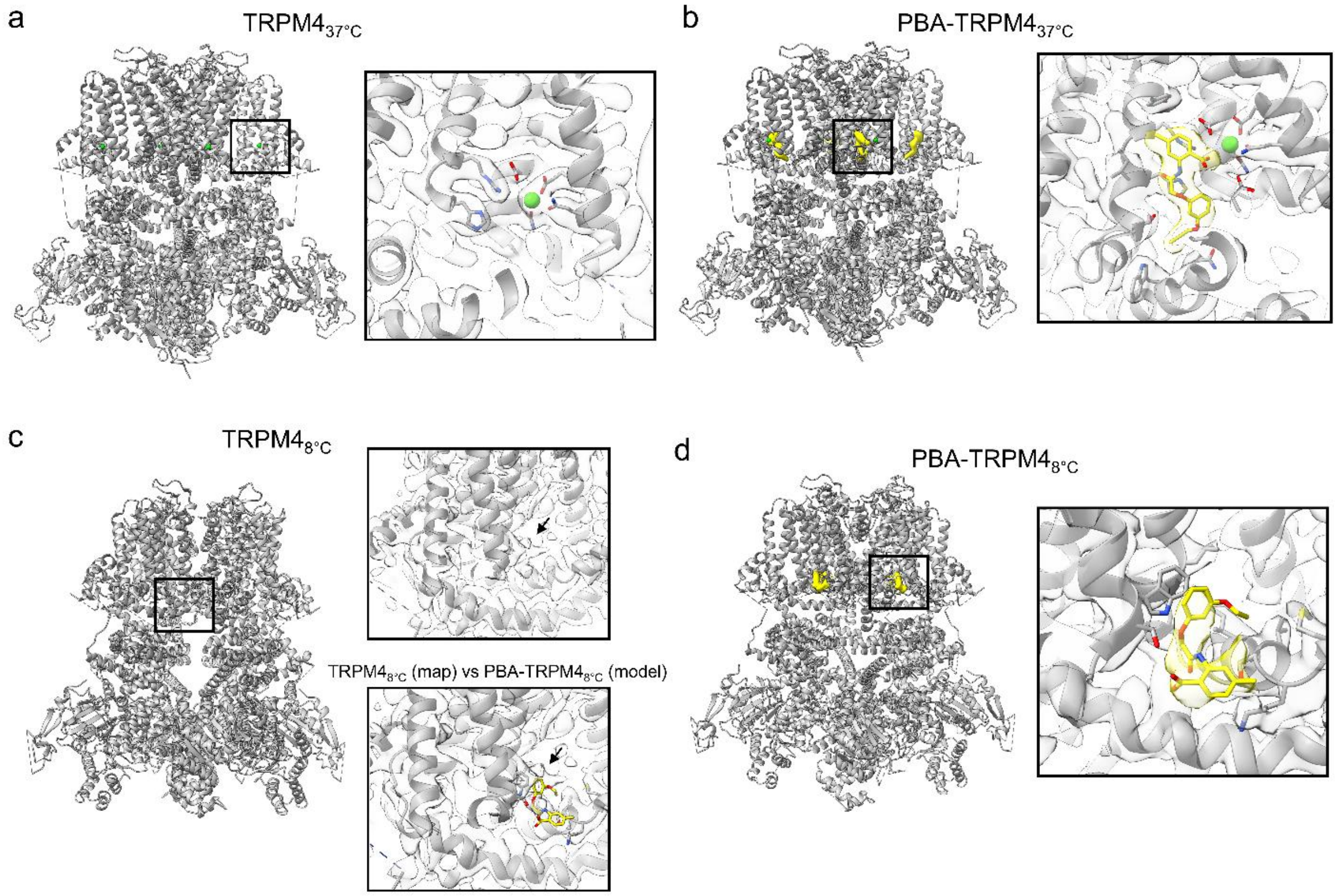

Supplementary Figure 7: **Cryo-EM density surrounding PBA-binding sites and neighbouring residues. (a)** TRPM4 at 37 °C in the absence of PBA. The boxed region highlights the PBA-binding region observed in the ligand-bound structure. The enlarged view shows density for neighbouring residues and the bound Ca^{2+} ion, but no corresponding density for PBA. **(b)** PBA-bound TRPM4 at 37 °C. The enlarged view shows the cryo-EM density surrounding PBA and neighbouring residues within the S1–S4 binding site. **(c)** TRPM4 at 8 °C in the absence of PBA. The upper enlarged view shows a non-protein density close to the 8 °C PBA-binding pocket. The lower enlarged view shows an overlay with the PBA-bound model, indicating that this density does not overlap with PBA and likely corresponds to a lipid-like species. **(d)** PBA-bound TRPM4 at 8 °C. The enlarged view shows the cryo-EM density surrounding PBA and neighbouring residues within the transmembrane inhibitor-binding pocket.
