# Supplementary material for: Temperature-dependent ligand relocation reveals plasticity of TRPM4 inhibition
Source: bioRxiv. 2026 May 14:2026.05.13.724805. Preprint. [Version 1] doi: 10.64898/2026.05.13.724805 (PMC13192758; doi:10.64898/2026.05.13.724805)
Supplement: Supplementary file 1 [file NIHPP2026.05.13.724805v1-supplement-1.pdf]

bioRxiv preprint doi: https://doi.org/10.64898/2026.05.13.724805; this version posted May 14, 2026. The copyright holder for this preprint (which was not certified by peer review) is the author/funder, who has granted bioRxiv a license to display the preprint in perpetuity. It is made available under a CC-BY 4.0 International license.

Supplementary Figures

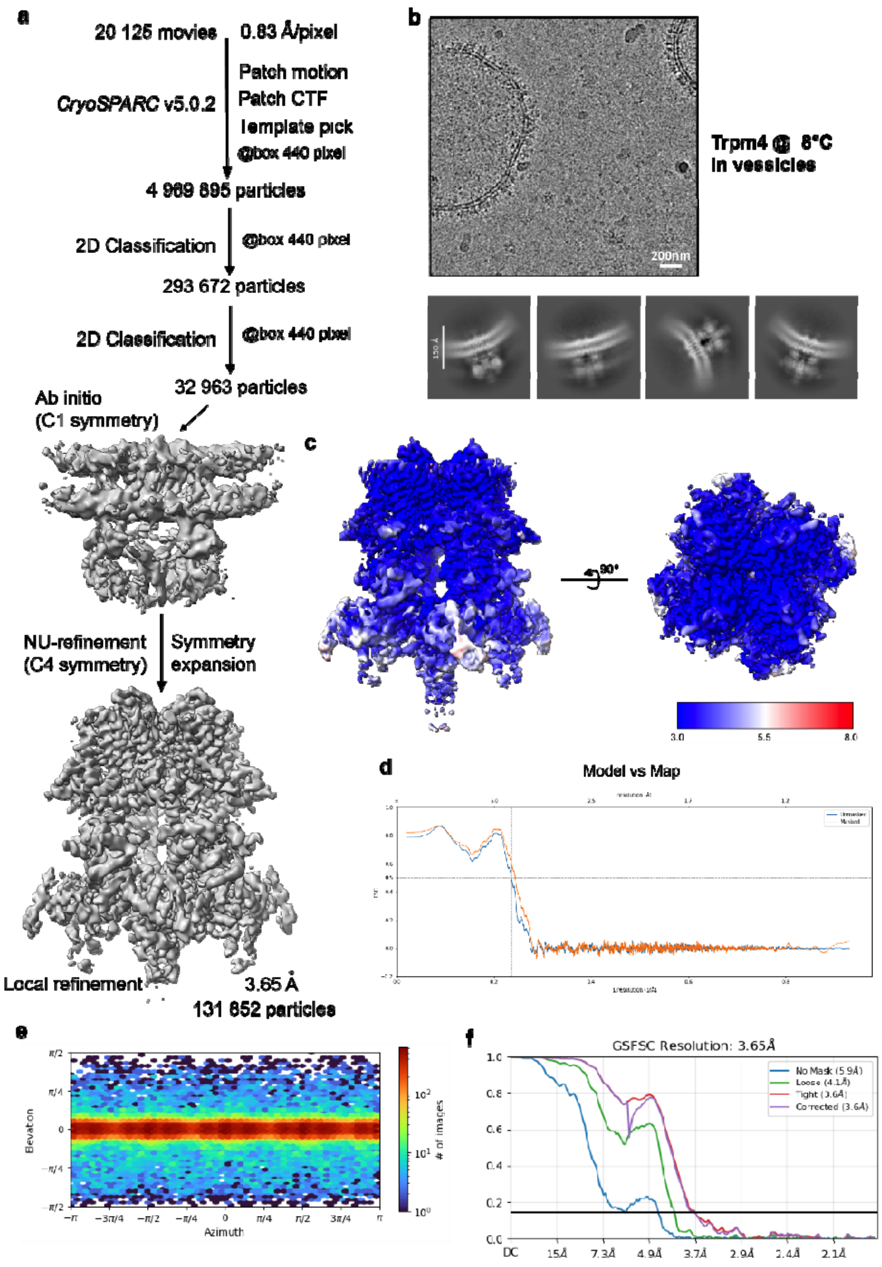

Supplementary Figure 1: **Cryo-EM data processing workflow and map resolution** (a) The image processing workflow of $\text{TRPM4}_{\text{vesicle}}$. (b) micrograph and 2D classes (c) Local resolution (d) Model vs map FSC curves. (e) particle direction distribution. (f) FSC curves.

bioRxiv preprint doi: https://doi.org/10.64898/2026.05.13.724805; this version posted May 14, 2026. The copyright holder for this preprint (which was not certified by peer review) is the author/funder, who has granted bioRxiv a license to display the preprint in perpetuity. It is made available under a CC-BY 4.0 International license.

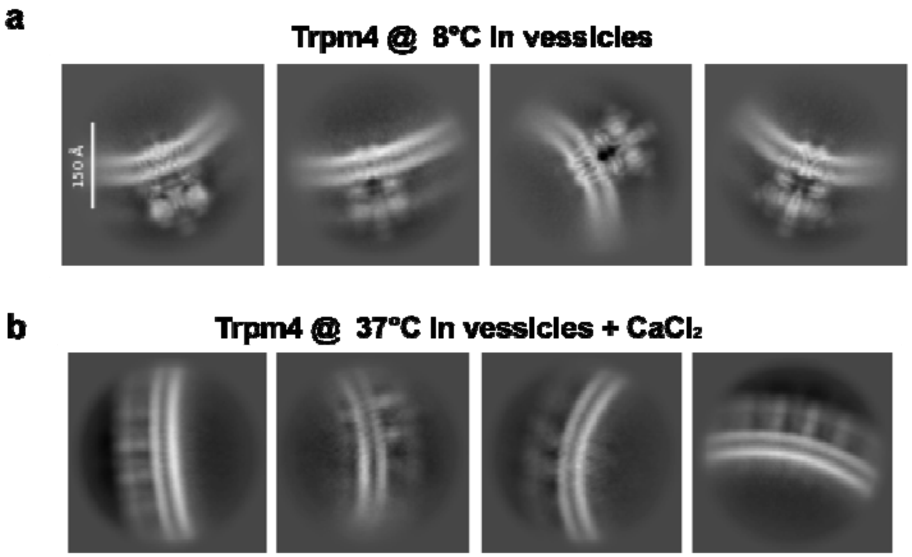

Supplementary Figure 2: **Comparison of vesicle-derived TRPM4 2D classes at 8□°C and 37□°C**. (a) Representative 2D class averages of TRPM4 particles derived from whole-cell vesicles prepared at 8□°C, showing well-defined structural features.(b) Representative 2D class averages of TRPM4 particles derived from whole-cell vesicles prepared at 37□°C, showing blurred and poorly resolved features.

bioRxiv preprint doi: https://doi.org/10.64898/2026.05.13.724805; this version posted May 14, 2026. The copyright holder for this preprint (which was not certified by peer review) is the author/funder, who has granted bioRxiv a license to display the preprint in perpetuity. It is made available under a CC-BY 4.0 International license.

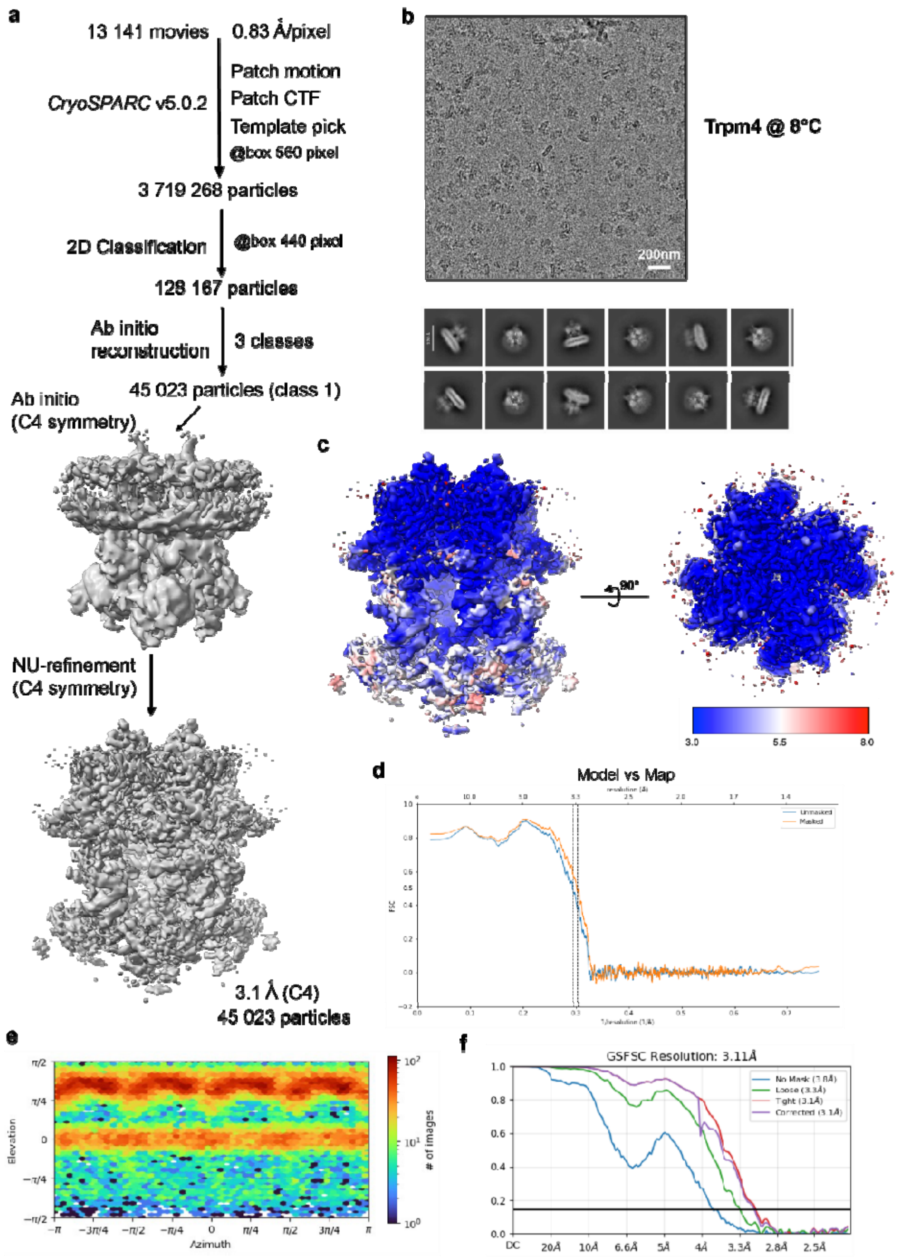

Supplementary Figure 3: **Cryo-EM data processing workflow and map resolution** (a) The image processing workflow of $TRPM4_{apo, 8\,°C}$. (b) micrograph and 2D classes (c) Local resolution (d) Model vs map FSC curves. (e) particle direction distribution. (f) FSC curves.

bioRxiv preprint doi: https://doi.org/10.64898/2026.05.13.724805; this version posted May 14, 2026. The copyright holder for this preprint (which was not certified by peer review) is the author/funder, who has granted bioRxiv a license to display the preprint in perpetuity. It is made available under a CC-BY 4.0 International license.

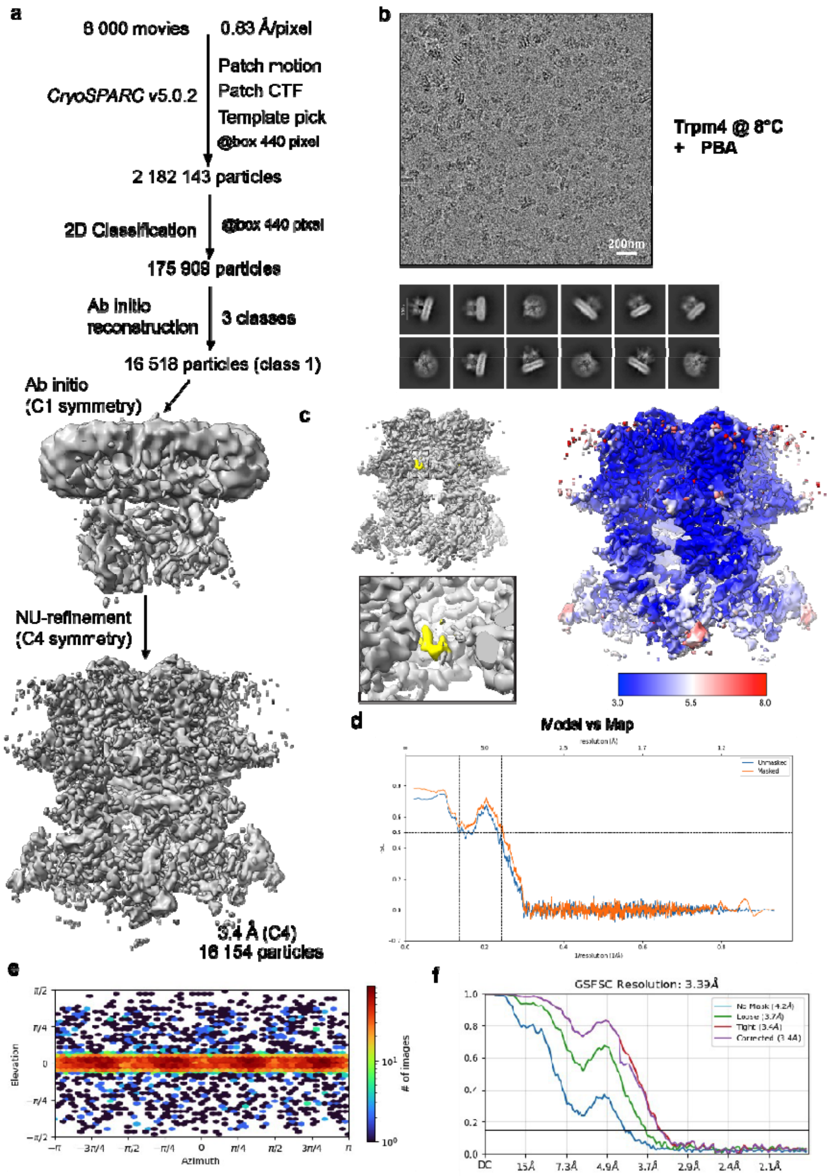

Supplementary Figure 4: **Cryo-EM data processing workflow and map resolution** (a) The image processing workflow of TRPM4$_{PBA,\ 8\ °C}$. (b) micrograph and 2D classes (c) Local resolution and PBA density (yellow) (d) Model vs map FSC curves. (e) particle direction distribution. (f) FSC curves.

bioRxiv preprint doi: https://doi.org/10.64898/2026.05.13.724805; this version posted May 14, 2026. The copyright holder for this preprint (which was not certified by peer review) is the author/funder, who has granted bioRxiv a license to display the preprint in perpetuity. It is made available under a CC-BY 4.0 International license.

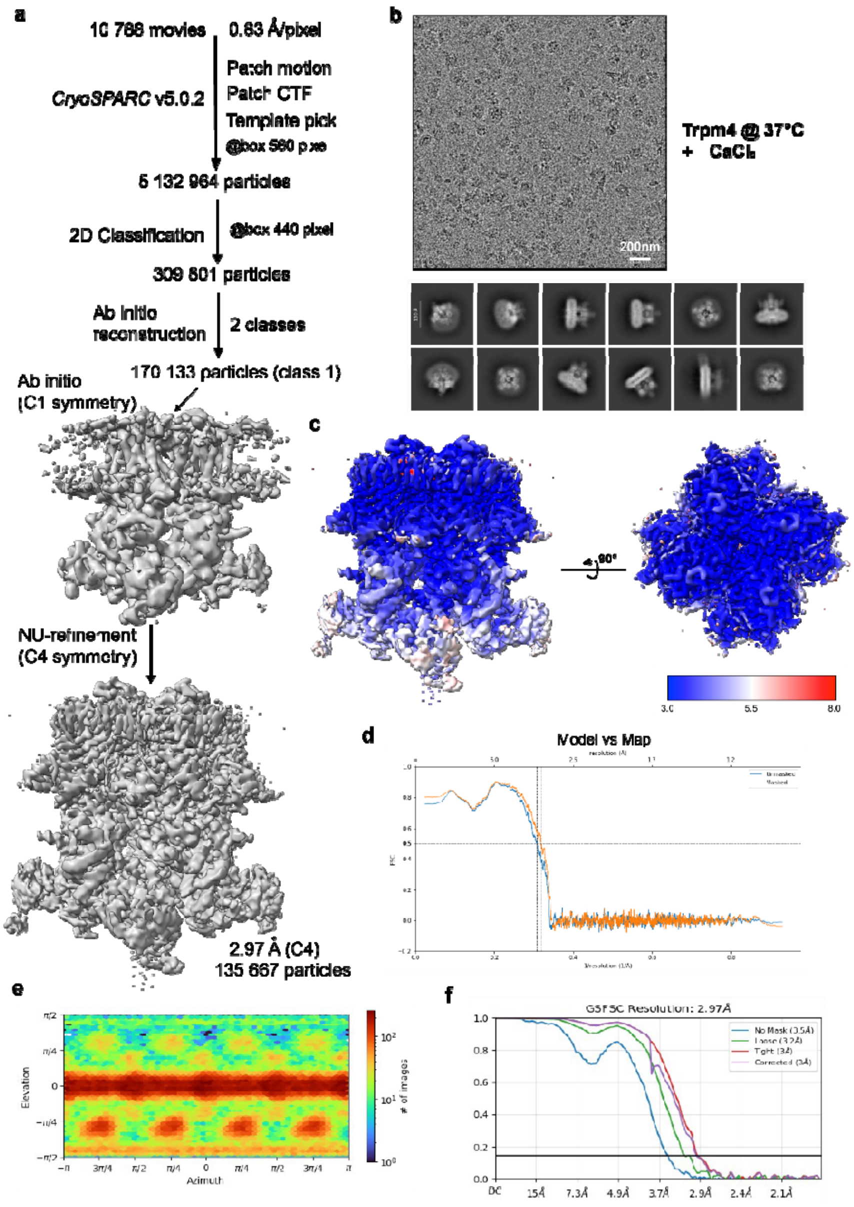

Supplementary Figure 5: **Cryo-EM data processing workflow and map resolution** (a) The image processing workflow of $TRPM4_{apo,\ 37\ °C}$. (b) micrograph and 2D classes (c) Local resolution (d) Model vs map FSC curves. (e) particle direction distribution. (f) FSC curves.

bioRxiv preprint doi: https://doi.org/10.64898/2026.05.13.724805; this version posted May 14, 2026. The copyright holder for this preprint (which was not certified by peer review) is the author/funder, who has granted bioRxiv a license to display the preprint in perpetuity. It is made available under a CC-BY 4.0 International license.

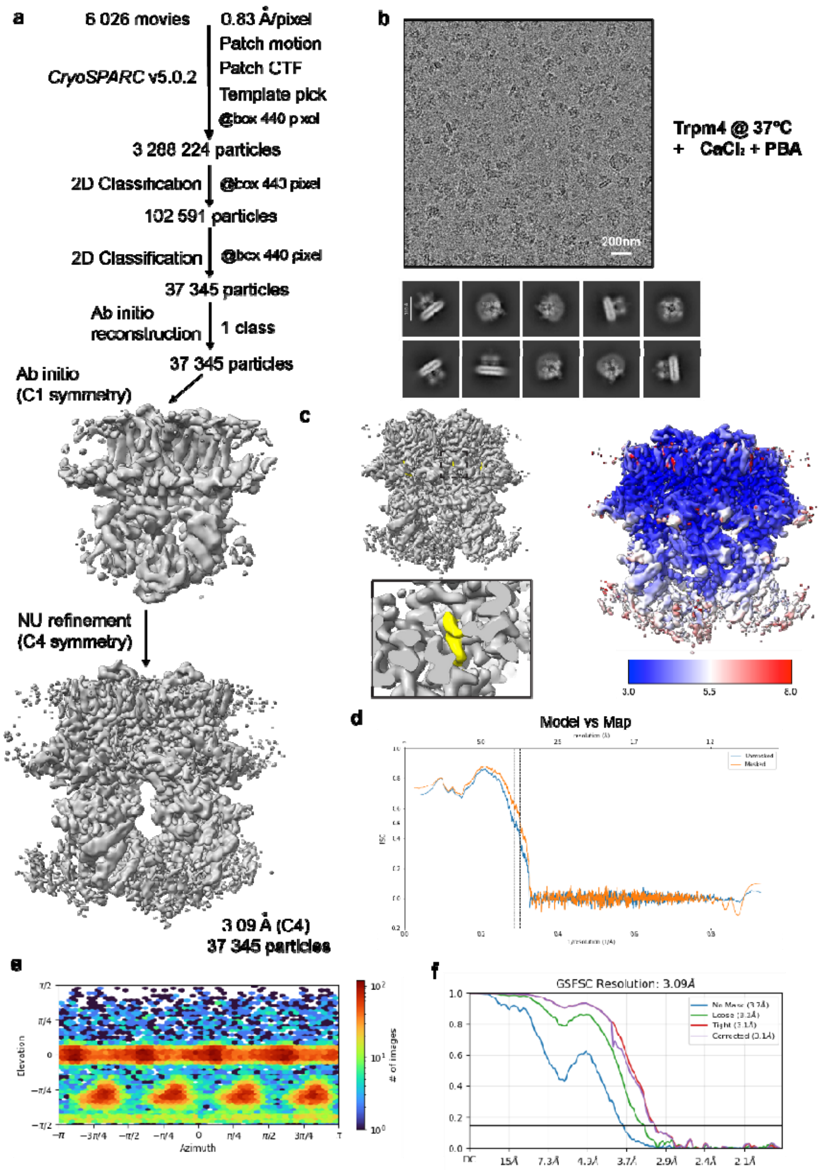

Supplementary Figure 6: **Cryo-EM data processing workflow and map resolution** (a) The image processing workflow of $TRPM4_{PBA,\ 37\ °C}$. (b) micrograph and 2D classes (c) Local resolution and PBA density (yellow) (d) Model vs map FSC curves. (e) particle direction distribution. (f) FSC curves.

bioRxiv preprint doi: https://doi.org/10.64898/2026.05.13.724805; this version posted May 14, 2026. The copyright holder for this preprint (which was not certified by peer review) is the author/funder, who has granted bioRxiv a license to display the preprint in perpetuity. It is made available under a CC-BY 4.0 International license.

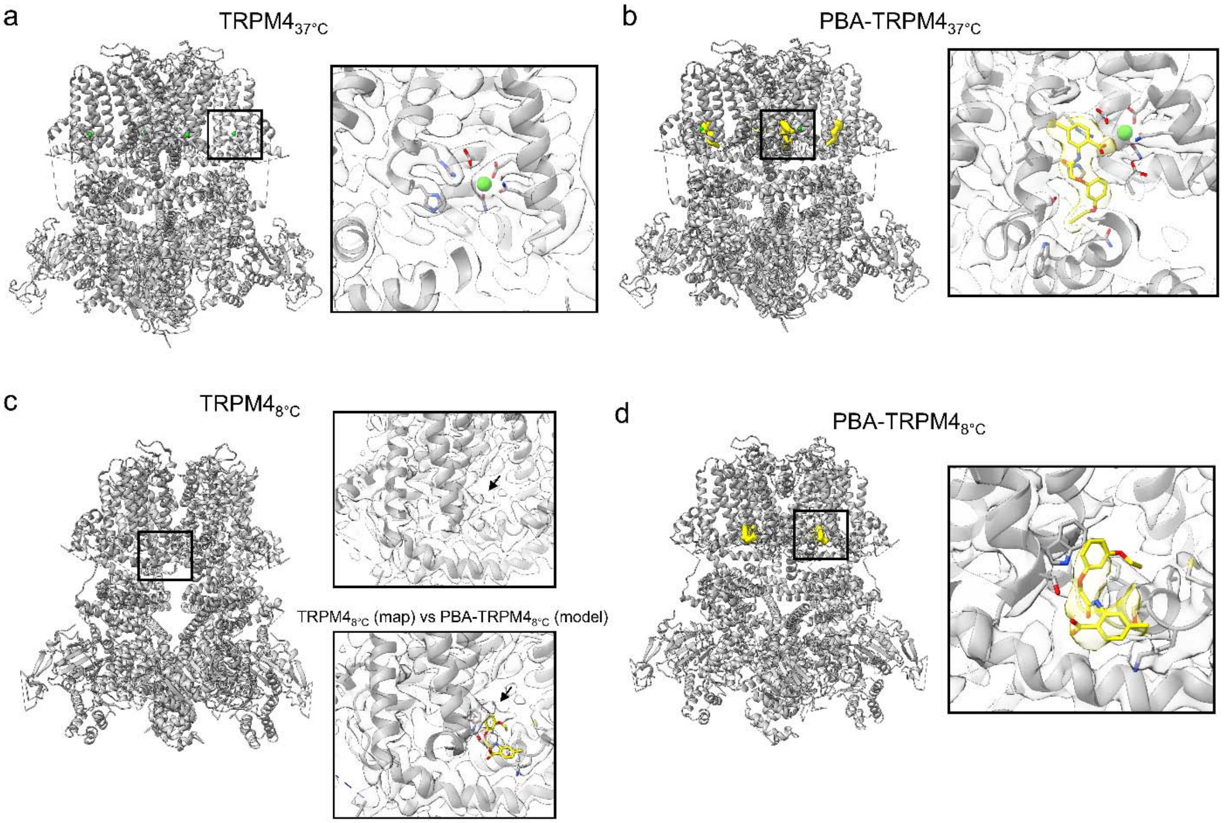

Supplementary Figure 7: **Cryo-EM density surrounding PBA-binding sites and neighbouring residues.** **(a)** TRPM4 at 37 °C in the absence of PBA. The boxed region highlights the PBA-binding region observed in the ligand-bound structure. The enlarged view shows density for neighbouring residues and the bound Ca^{2+} ion, but no corresponding density for PBA. **(b)** PBA-bound TRPM4 at 37 °C. The enlarged view shows the cryo-EM density surrounding PBA and neighbouring residues within the S1–S4 binding site. **(c)** TRPM4 at 8 °C in the absence of PBA. The upper enlarged view shows a non-protein density close to the 8 °C PBA-binding pocket. The lower enlarged view shows an overlay with the PBA-bound model, indicating that this density does not overlap with PBA and likely corresponds to a lipid-like species. **(d)** PBA-bound TRPM4 at 8 °C. The enlarged view shows the cryo-EM density surrounding PBA and neighbouring residues within the transmembrane inhibitor-binding pocket.
